# Laser-induced breakdown spectroscopy for the measurement of air humidity using spectral line intensity ratio

**DOI:** 10.1039/d5ra05015d

**Published:** 2025-07-30

**Authors:** Jianyong Cen, Xin Wang, Zeguang Li, Ni An, Jinjun Zhang

**Affiliations:** a College of Physics and Electronic Engineering, Shanxi Normal University Taiyuan 030031 China zhangjinjun@sxnu.edu.cn

## Abstract

Laser-induced breakdown spectroscopy (LIBS) is widely used in air environment. Humidity is an important parameter in the air environment, and its variation can affect the accuracy of LIBS. Therefore, measurement of humidity is very necessary when performing LIBS applications. We present a method for humidity measurement using laser-induced breakdown spectroscopy. The intensity ratio between spectral lines was used to establish the relationship with relative humidity. The relative humidity has a linear relationship with the intensity ratio of Hα 656.3 nm to N II 463.0 nm, N II 500.5 nm, N I 746.8 nm and O I 777.4 nm. The linearity can exceed 99.9%. The linear relationship was also used to measure the air humidity, and the measurement results were accurate. This study confirms the feasibility of using laser-induced breakdown spectroscopy to measure humidity. The advantage of this method is that when using LIBS applications, no other instruments are required to measure humidity.

## Abstract 2

Laser-induced breakdown spectroscopy for the measurement of air humidity by using the ratio of spectral line intensities, which can enhance the measurement accuracy.

## Graphical abstract

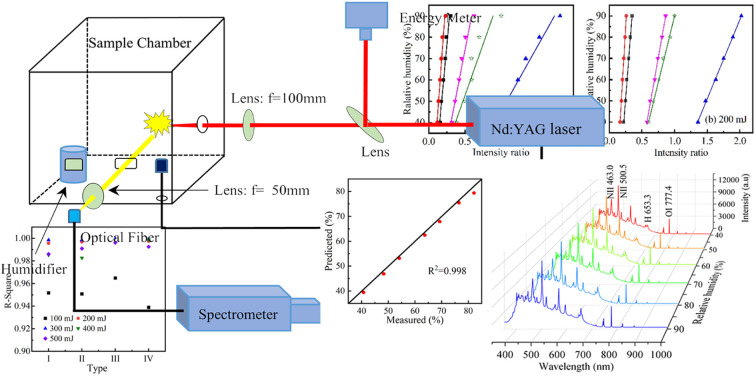

## 1. Introduction

Laser-induced breakdown spectroscopy (LIBS) is an analysis technique of plasma emission spectrometry.^1,2^ It works by using a focused pulsed laser on a solid, liquid or gas to vaporize the sample momentarily and produce a plasma of tens of thousands of degrees.^3^ Various substances in the sample are decomposed into basic elements. The excited atoms or ions can emit strong spectral lines with characteristic wavelengths.^4,5^ The intensities of these spectral lines are closely related to the content of the element.^6^ Therefore, LIBS can be used for the type identification and content measurement of the elements.^7–10^ With the advantages of portable equipment, simple sample preparation process (or no sample preparation), online analysis, low cost, fast analysis speed, and quantitative measurement function, LIBS has been widely used in air environment monitoring.^11–15^

The atmosphere, as an important material foundation for human living environment, is a combination of dry air and water vapor. Although water vapor is only a small part of the atmosphere, changes in its content have important effects on the environment. Humidity is a physical quantity that represents the dryness of the atmospheric and is an important parameter for characterizing the air environment.^16^ Since LIBS is widely used in different seasons, different regions, different experimental sites and other air environments with large differences in humidity, humidity has a great influence on the measurement results of LIBS.^17–23^ The effect of relative humidity on the spectrum of laser-induced air plasma was reported in some literature. Yalçin *et al.* found that the effect of humidity on the temperature and electron density of the laser-induced air plasma is negligible.^24^ Lazic *et al.* found a strong dependence of the emission spectra of hydrogen atoms on humidity when analyzing explosives and organic residues with LIBS.^25^ Ding *et al.* studied the emission intensity of oxygen, nitrogen and hydrogen lines under different air humidities.^26^ There is a linear relationship between the intensity of the emission line of Hα and the relative humidity of the air. Sun *et al.* also reported the similar results.^27^ Lei *et al.* investigated the influence of relative humidity on the laser-induced air plasma by varying humidity at different laser irradiances.^28^ The intensity of Hα increases with the humidity at a high laser energy. Liu *et al.* investigated the influence of ambient humidity on LIBS spectra.^29^ They found that the Hα 656.3 nm intensity increased with humidity, whereas the variation of the sample Cu I line intensity with humidity depended on the laser energy. Qin *et al.* analyzed the moisture absorption of aluminum phosphate pollution on the surface of an insulator by LIBS technology.^30^ They found that the normalization method can eliminate the influence of moisture on LIBS detection in the humidity range of 70–85%. So far, these studies have focused on the impact of humidity on LIBS technology,^31–33^ but few studies have used LIBS technology to measure humidity.

In this work, the emission spectra of laser induced air plasma at different laser energies and relative humidities are obtained. The relationship between the intensity of some characteristic lines and relative humidity under different laser energies is studied in detail. A linear relationship between the ratio of hydrogen line intensity to oxygen and nitrogen line intensity and relative humidity is innovatively established. The accuracy of using the method to measure relative humidity is verified. This study can provide a new method for measuring ambient air humidity, and may also be applied to the measurement of air humidity in extreme environments.

## 2. Experiment setup

**Fig. 1:**
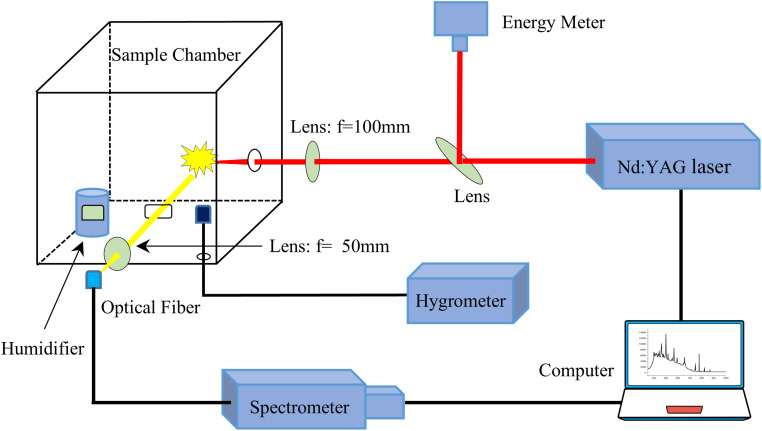
Schematic diagram of the experimental setup.

Fig. 1 shows a schematic diagram of the experimental setup for showing the formation and emission spectrum collection of laser induced air plasma. The experimental setup is mainly composed of laser source, air plasma formation area and collection device. The light source is a 1064 nm beam of Q-switched Nd:YAG pulse laser. The maximum output energy is 500 mJ per pulse. The repetition rate can be adjusted from 1 to 10 Hz. The pulse width is 8 ns. The laser beam is divided into two beams by the beam splitter. A small part of the laser beam is monitored by using a laser precision energy meter, the other part is focused by a lens with a focal length of 100 mm. Air is broken down at the focal point of the lens to form a high temperature air plasma, which occurs in a cube container with a side length of 50 cm. After the air plasma luminescence is generated, it is focused by a lens with a focal length of 50 mm, and then recorded by a fiber optic spectrometer and transmitted the spectral data to a computer. The spectrometer has a spectral range of 200–1100 nm. The wavelength resolution is 0.25 nm. The integration time is 4 ms and the delay time is 0 ms for each spectrum. In order to reduce the standard deviation, each spectrum was obtained by averaging 5 shots. The ambient humidity is controlled by an adjustable humidifier. The adjustable humidifier can automatically display and regulate the humidity of the surrounding environment. Its measurement accuracy is ±1.5%. The humidity adjustment range is from 40% to 90%. The stabilization time is approximately 1 minute. The experimental ambient temperature is 17 °C and the initial humidity is set to 40 RH%. The relative humidity can also be measured by a hygrometer.

## 3. Results

**Fig. 2:**
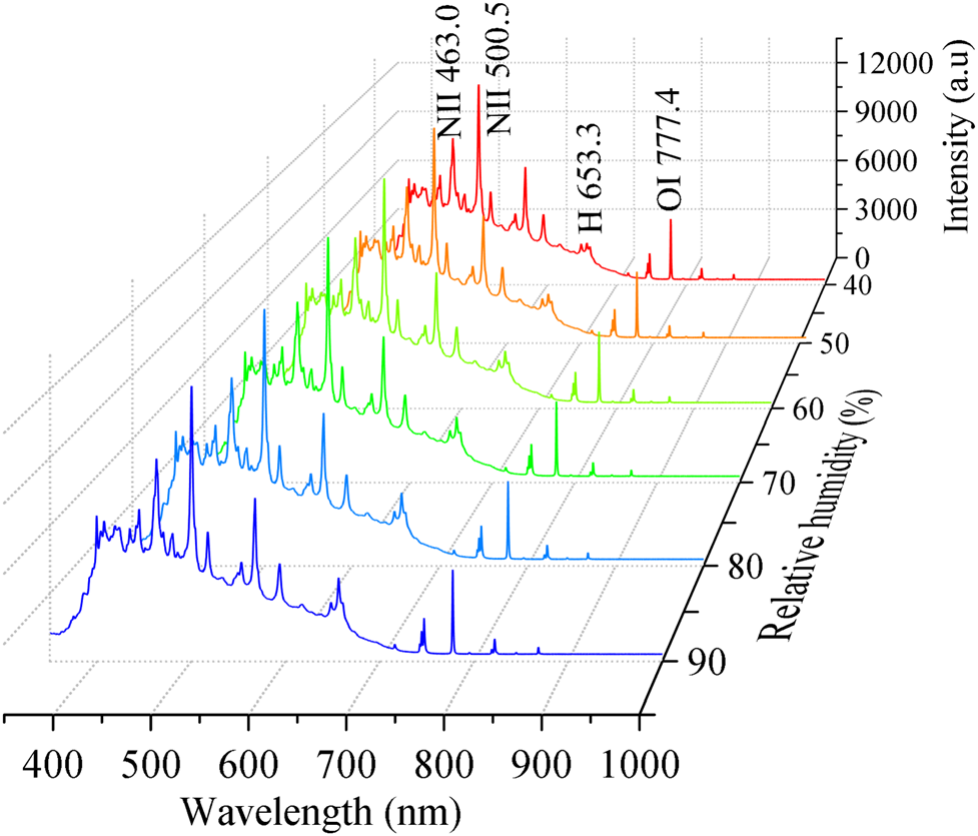
The spectra of laser-induced air plasma at different relative humidities.

Fig. 2 shows the emission spectra of laser-induced air plasma at relative humidities ranging from 40 to 90 RH%. The laser energy is 300 mJ. The emission spectrum is mainly composed of continuous spectrum and linear spectrum. Since N, O and H are the main elements in the air mixed with water vapor, the main spectral lines are N I, N II, O I, and H in Fig. 2. Most of the spectral lines of neutral atoms are distributed in the near infrared spectrum range of 700–900 nm, while the ion spectral lines are distributed in the visible region of 350–600 nm.^34^ The intensity of Hα 656.3 nm is relatively weak at the relative humidity of 40%. But at the relative humidity of 90%, it is very strong. It can be seen that the intensity of the Hα 656.3 nm increases with increasing relative humidity. In contrast, the intensities of the spectral lines of other elements have little relationship with relative humidity. This is due to the fact that air is composed of 78% nitrogen, 21% oxygen, 1% rare gases and impurities. The source of N and O elements is relatively stable, so the increase of water vapor will not have a significant effect on it.

**Fig. 3:**
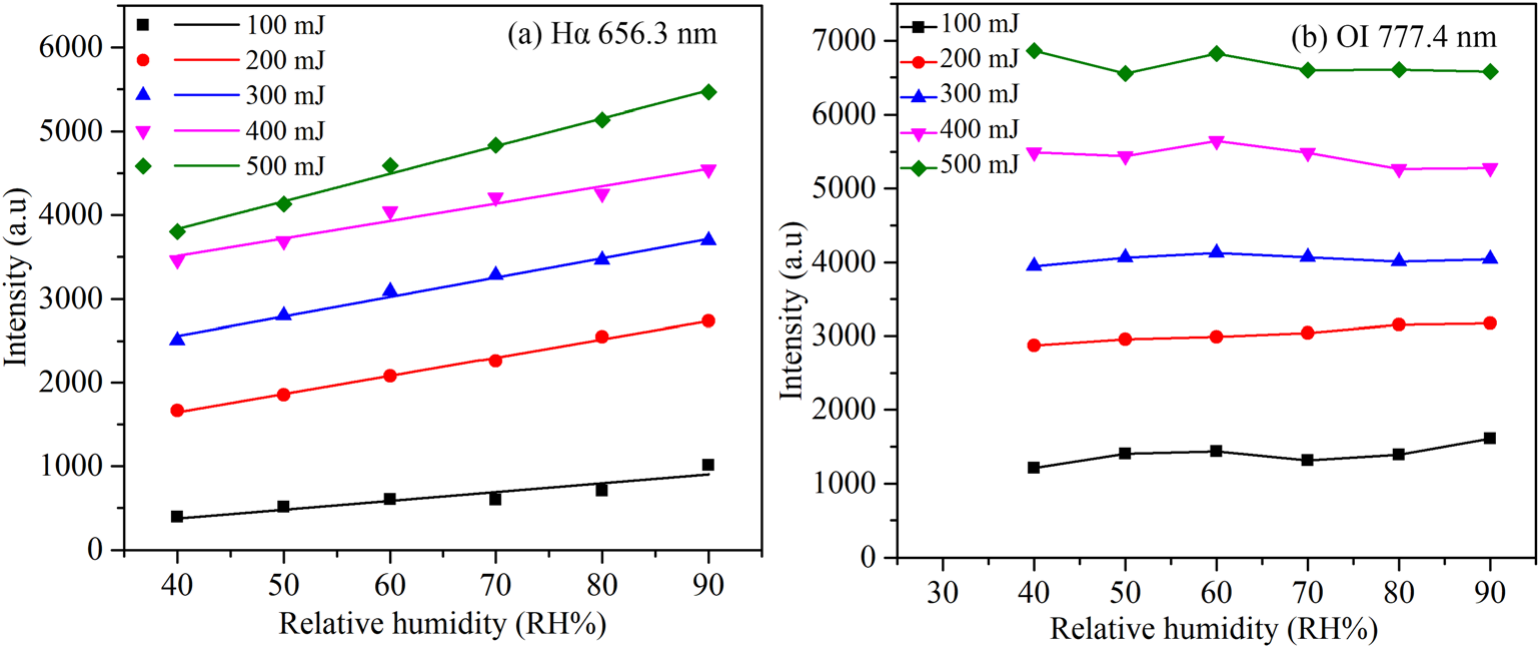
(a) Linear fitting of Hα line intensity with relative humidity at different energies. (b) The change of OI linear intensity with relative humidity at different energies.

In order to further study the relationship between the spectral line intensity and relative humidity, Fig. 3 shows the variation of the intensities of the Hα 656.3 nm and O I 777.4 nm with relative humidity. It is known that in LIBS detection, when the laser energy is too high, certain spectral lines (particularly Hα) may experience saturation. In our experiment, there was no saturation in the intensity of all spectral lines at high energy by adjusting the distance between the air plasma point and the spectrometer probe. The spectrometer measures a maximum intensity of 16, 000, but the Hα line is only 7, 000 at 500 mJ energy. It can be seen in Fig. 3(a) that the intensity of Hα 656.3 nm increases linearly and has high linearity with the increase of relative humidity at different energies. However, the intensity of O I lines has no obvious correlation with relative humidity at different energies, as shown in Fig. 3(b). Similarly, the intensity of the N II and N I lines is not significantly correlated with relative humidity.

There is a linear relationship between Hα line intensity and relative humidity, which is consistent with previous studies.^27–29^ This also indicates that it is feasible to use air spectrum to measure relative humidity. However, the laser energy even at the set value, there is a certain fluctuation, which will lead to a difference in the intensity of the Hα 656.3 nm even at the same laser energy. If relative humidity is measured directly by the intensity of the Hα 656.3 nm, the stability of the measurement results is not high enough. When performing a linear fit using the Hα 656.3 nm line intensity and humidity at 100 mJ energy, the *R*-square value is only 0.83. We found that the value of the intensity ratio of Hα 656.3 nm to other elements is almost unaffected by fluctuation in laser energy. Therefore, we proceeded to investigate the relationship between the intensity ratio and relative humidity.

**Fig. 4:**
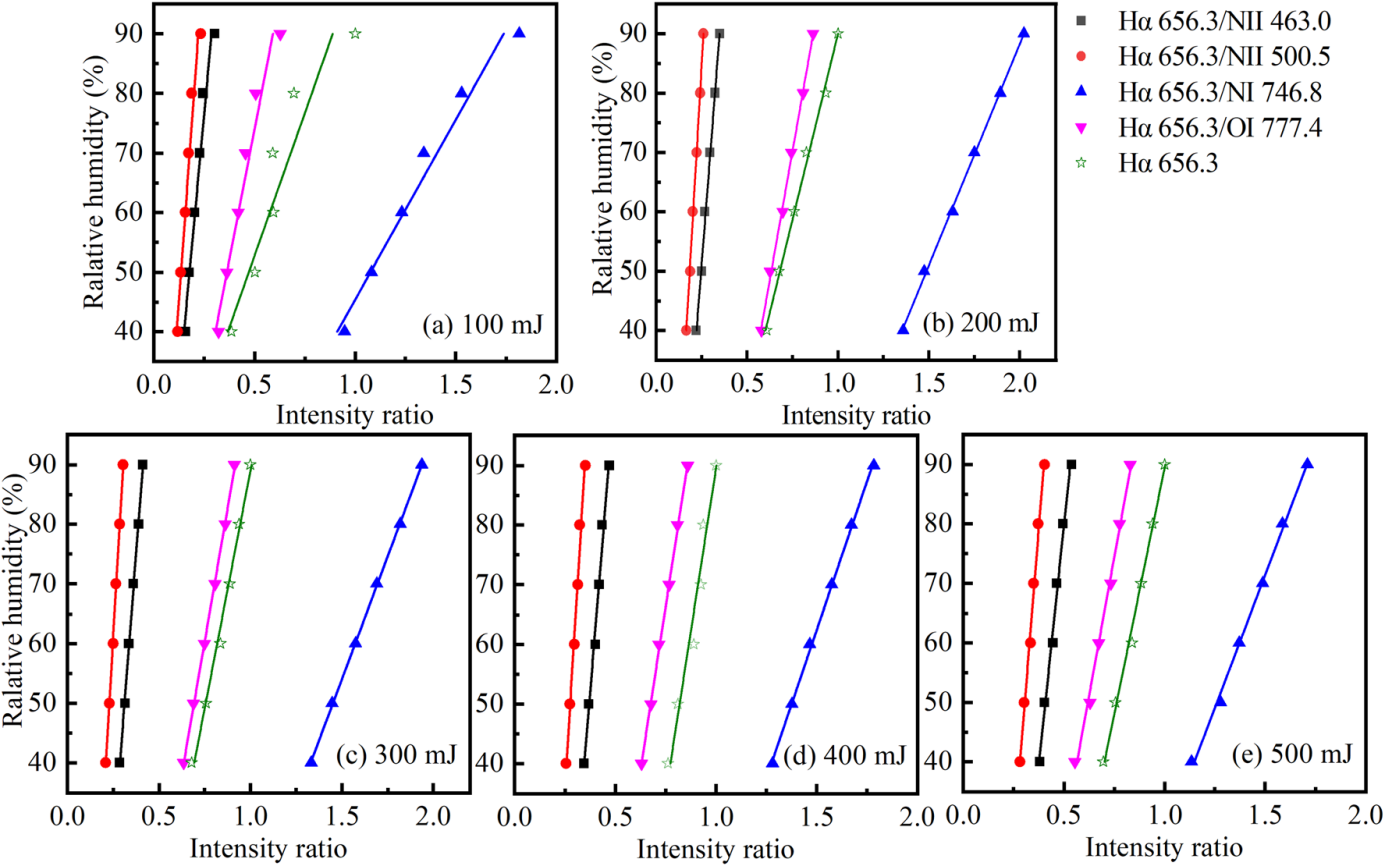
Relationship between intensity ratio and relative humidity at different energies.

**Fig. 5:**
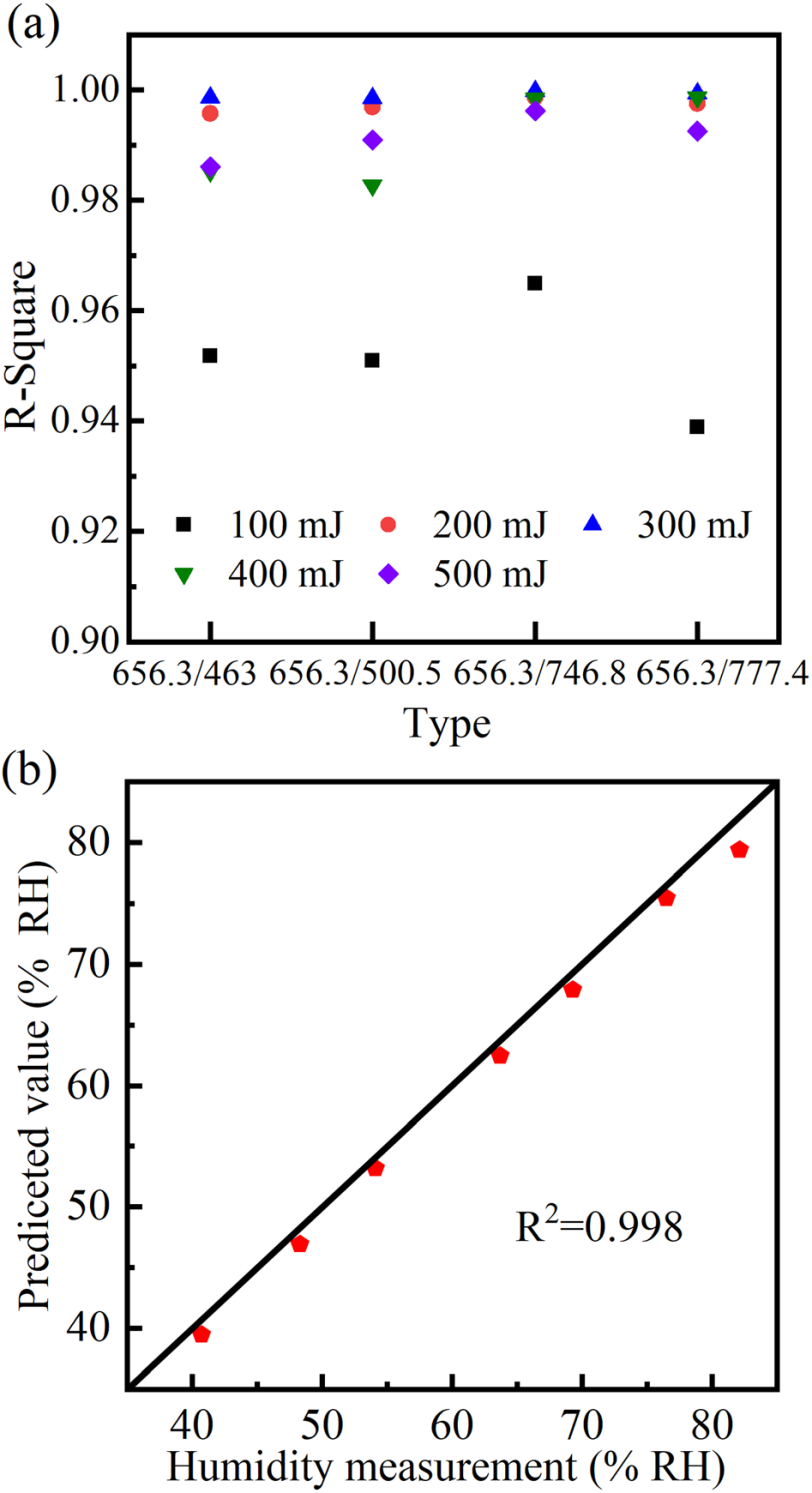
(a) *R*-Square of the fitted lines at different energies. (b) Comparison of the humidity measurement.

Fig. 4 shows the intensity ratio of the Hα 656.3 nm to other four spectral lines (N II 463.0 nm, N II 500.5 nm, N I 746.8 nm and O I 777.4 nm) as a function of relative humidity at different energies. It can be seen that there is a linear relationship between the intensity ratio and relative humidity at five laser energies. The fitted curve function is *y* = *a* + *bx*. *y* Represents the relative humidity. *x* Represents the intensity ratio. *R*-Square is an important measure of goodness of fit, with a higher value indicating a better fit. The closer the *R*-square is to 1, the higher the goodness of fit. Fig. 5(a) shows the *R*-square of the fitted lines. At the laser energy of 100 mJ, the fitting results are not very good. The *R*-square of the function fitted from the intensity ratio of Hα 656.3 nm and O I 777.4 nm is only 0.94. The maximum value of *R*-square is 0.965, which comes from the fitted straight line using the intensity ratio of Hα 656.3 nm and N I 746.8 nm. The main reason is that the intensity of the hydrogen lines is unstable at the 100 mJ energy. The laser did not ionize the air sufficiently, resulting in poor line shape, low intensity, and poor stability of the Hα 656.3 nm line. So, when the intensity is used for linear fitting, its effect is not very good.

In contrast, at the laser energies of 200 mJ and 300 mJ, the *R*-square values of the four fitted lines are all above 0.996. Especially when the laser energy is 300 mJ, the *R*-square of the fitted line from the intensity ratio of Hα 656.3 nm and NI 746.8 nm can reach 0.9997. The *R*-square is very close to 1, indicating that the relationship between relative humidity and intensity ratio is a linear function. With this linear function, the relative humidity of air can be obtained by measuring the intensity ratio of the spectral lines using the LIBS technique.

The use of the spectral line intensity ratio can decrease the instability of laser energy on the result of measurement. In Fig. 4, at five laser energies, the slopes of the linear function (using the intensity ratio of Hα 656.3 nm to O I 777.4 nm) are 0.0057, 0.0058, 0.0056, 0.0056 and 0.0054, respectively. It can be seen that the laser energy has little effect on the slope of this function. In Fig. 3, there is also a linear relationship between Hα line intensity and relative humidity. But under different laser energies, the variation of the slope is relatively large. It also indicates that the laser energy has a great influence on the spectral line intensity, but not on the ratio of spectral line intensity.

The relative humidity has been measured by using the intensity ratio, as shown in Fig. 5(b). We measured seven humidity values in the air and compared them with the measurement of hygrometer. It can be seen that our measurements are in good agreement with those of the hygrometer, indicating that it is feasible to use LIBS to measure the relative humidity of the air. An advantage of this method is that it does not require a very sophisticated spectrometer to accurately measure air humidity. The spectrometer used in this experiment has a minimum integration time of 4 ms and a wavelength resolution of 0.25 nm. Such spectrometers are common and inexpensive on the market. In addition, each spectrum was obtained by averaging 5 shots in this experiment. This means that the method does not need to repeat many times to take the average, and can get more accurate results.

## 4. Conclusions

Based on LIBS technology, a method is proposed to determine air relative humidity. Under different laser energies, the emission spectra of laser-induced air plasma at different relative humidities are obtained. The results show that Hα lines have a strong dependence on relative humidity. As the laser energy increases, the change in relative humidity makes the change in Hα line intensity more significant. The intensity ratio of the Hα line to the four characteristic spectral lines is selected to measure the relative humidity. At the laser energy of 300 mJ, the linear fit between spectral line intensity ratio and relative humidity is more than 99.9%. The experimental results demonstrate that it is feasible to measure the relative humidity of air by LIBS. In future work, we will investigate the accuracy of this method in extreme environments, such as high temperature and high pressure.

## Author contributions

The manuscript was written by J. C., X. W.; the experiments were carried out by all authors; the manuscript was review and editing by J. C., J. Z.

## Conflicts of interest

The authors declare no conflicts of interest.

## Data Availability

Data for this article are available at [Baidu Netdisk] at [https://pan.baidu.com/s/1qXk40ijoAv3kTsLYmjGsmg?pwd=kc6x].
